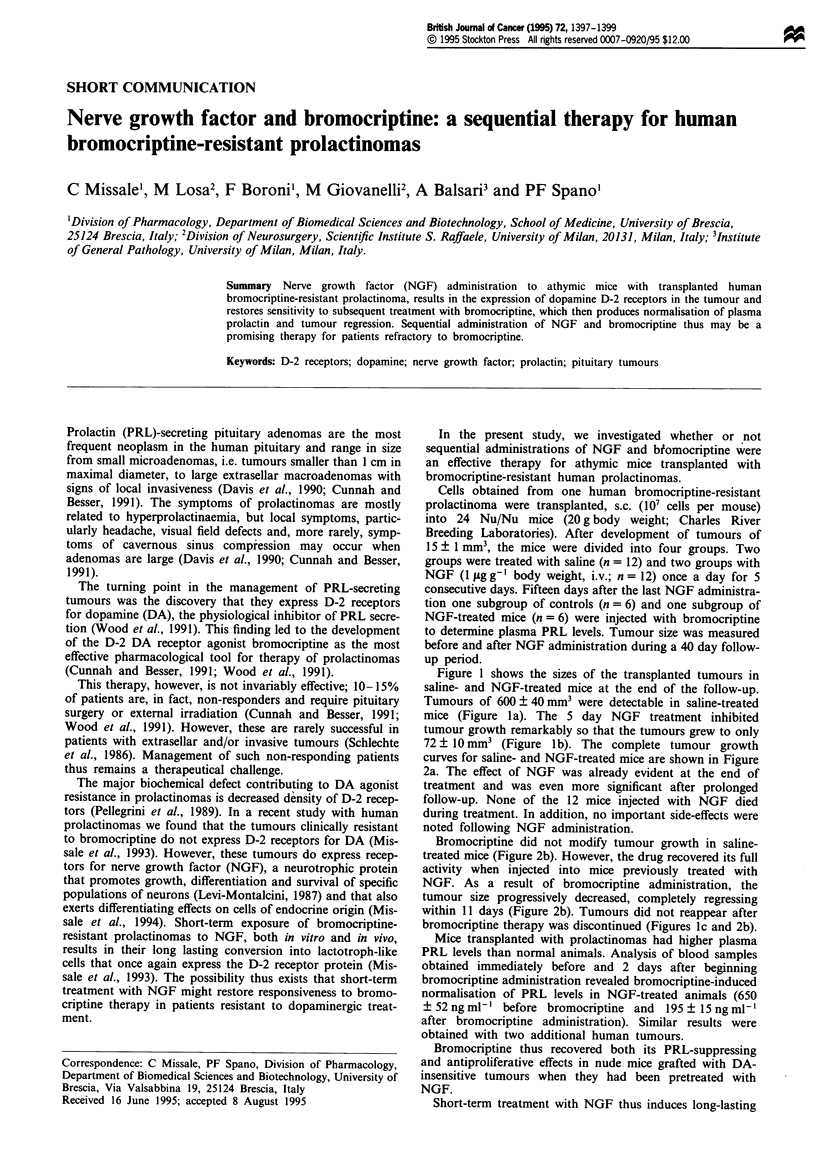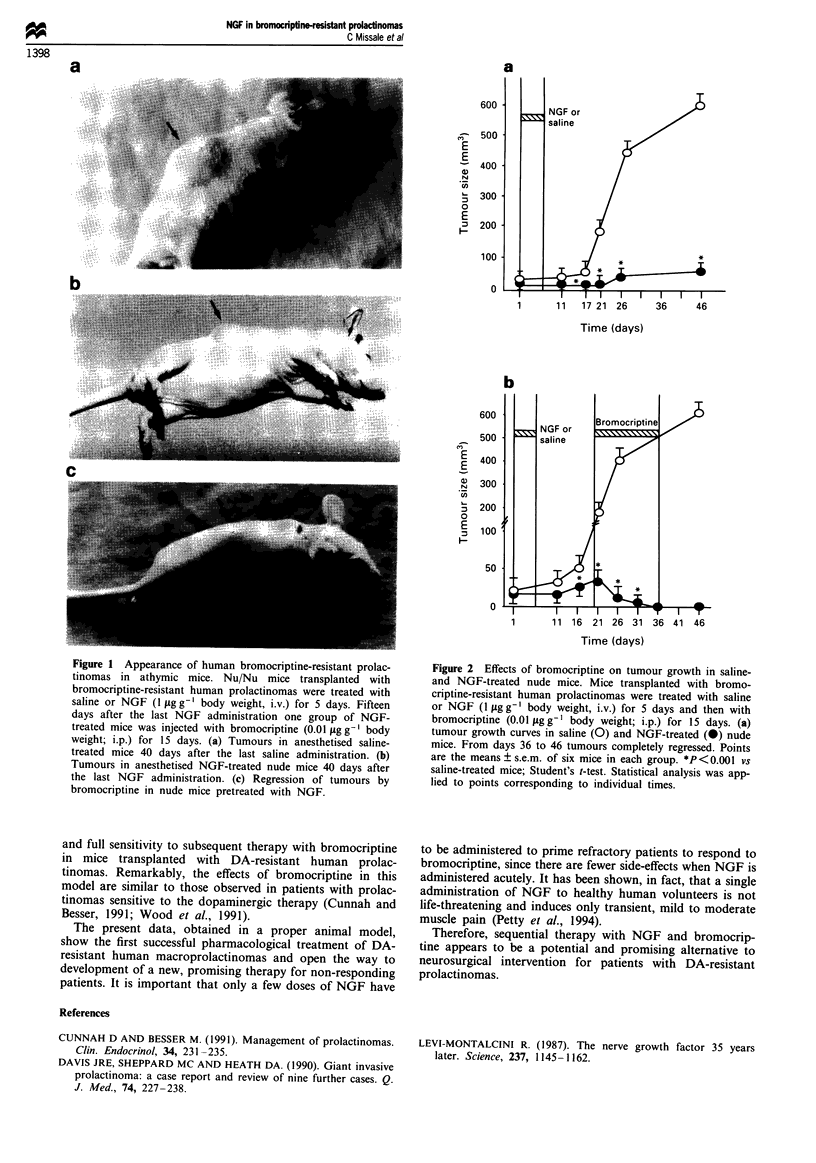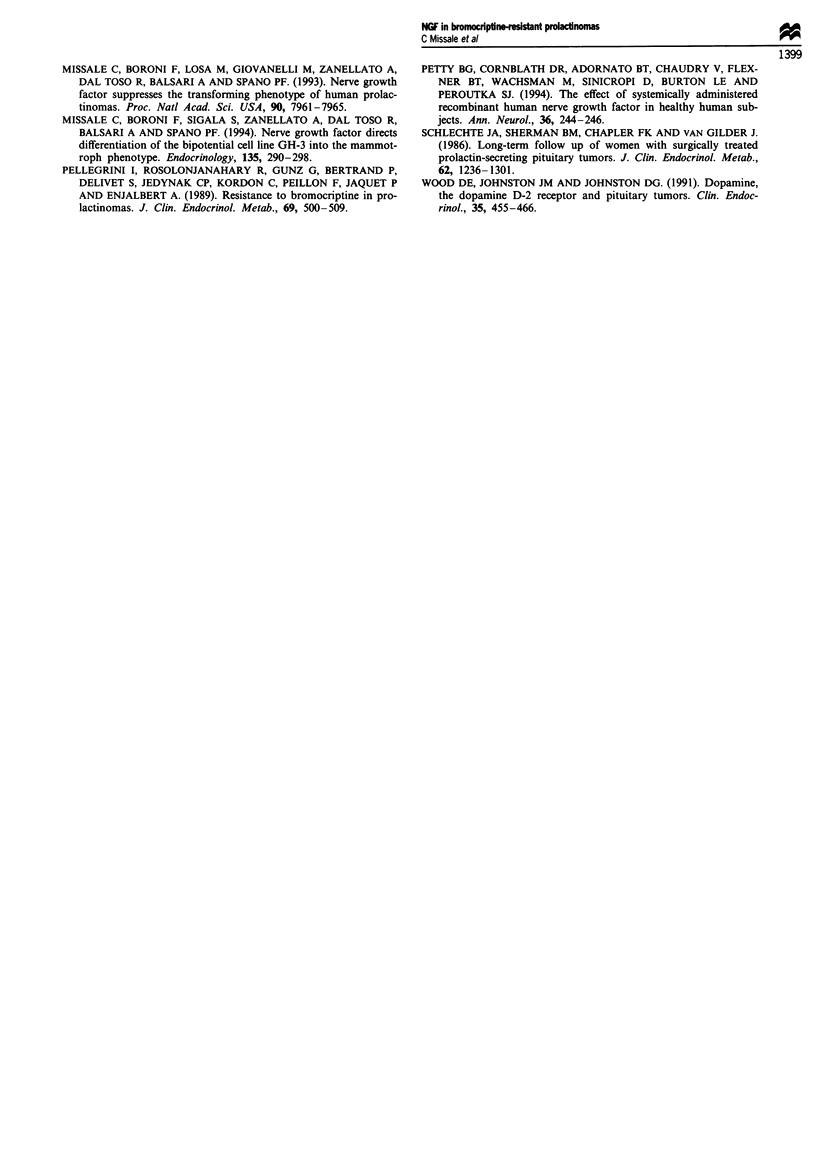# Nerve growth factor and bromocriptine: a sequential therapy for human bromocriptine-resistant prolactinomas.

**DOI:** 10.1038/bjc.1995.520

**Published:** 1995-12

**Authors:** C. Missale, M. Losa, F. Boroni, M. Giovanelli, A. Balsari, P. F. Spano

**Affiliations:** Department of Biomedical Sciences and Biotechnology, School of Medicine, University of Brescia, Italy.

## Abstract

**Images:**


British Journal of Cancer (1995) 72, 1397-1399
© 1995 Stockton Press All rights reserved 0007-0920/95 $12.00

SHORT COMMUNICATION

### Nerve growth factor and bromocriptine: a sequential therapy for human bromocriptine-resistant prolactinomas

C Missale[1], M Losa[2], F Boroni[1], M Giovanelli[2], A Balsari[3] and PF Spano[1]

[1]*Division of Pharmacology, Department of Biomedical Sciences and Biotechnology, School of Medicine, University of Brescia, 25124 Brescia, Italy;* [2]*Division of Neurosurgery, Scientific Institute S. Raffaele, University of Milan, 20131, Milan, Italy;* [3]*Institute of General Pathology, University of Milan, Milan, Italy.*

**Summary** Nerve growth factor (NGF) administration to athymic mice with transplanted human bromocriptine-resistant prolactinoma, results in the expression of dopamine D-2 receptors in the tumour and restores sensitivity to subsequent treatment with bromocriptine, which then produces normalisation of plasma prolactin and tumour regression. Sequential administration of NGF and bromocriptine thus may be a promising therapy for patients refractory to bromocriptine.

**Keywords:** D-2 receptors; dopamine; nerve growth factor; prolactin; pituitary tumours

Prolactin (PRL)-secreting pituitary adenomas are the most frequent neoplasm in the human pituitary and range in size from small microadenomas, i.e. tumours smaller than 1 cm in maximal diameter, to large extrasellar macroadenomas with signs of local invasiveness (Davis *et al.*, 1990; Cunnah and Besser, 1991). The symptoms of prolactinomas are mostly related to hyperprolactinaemia, but local symptoms, particularly headache, visual field defects and, more rarely, symptoms of cavernous sinus compression may occur when adenomas are large (Davis *et al.*, 1990; Cunnah and Besser, 1991).

The turning point in the management of PRL-secreting tumours was the discovery that they express D-2 receptors for dopamine (DA), the physiological inhibitor of PRL secretion (Wood *et al.*, 1991). This finding led to the development of the D-2 DA receptor agonist bromocriptine as the most effective pharmacological tool for therapy of prolactinomas (Cunnah and Besser, 1991; Wood *et al.*, 1991).

This therapy, however, is not invariably effective; 10-15% of patients are, in fact, non-responders and require pituitary surgery or external irradiation (Cunnah and Besser, 1991; Wood *et al.*, 1991). However, these are rarely successful in patients with extrasellar and/or invasive tumours (Schlechte *et al.*, 1986). Management of such non-responding patients thus remains a therapeutical challenge.

The major biochemical defect contributing to DA agonist resistance in prolactinomas is decreased density of D-2 receptors (Pellegrini *et al.*, 1989). In a recent study with human prolactinomas we found that the tumours clinically resistant to bromocriptine do not express D-2 receptors for DA (Missale *et al.*, 1993). However, these tumours do express receptors for nerve growth factor (NGF), a neurotrophic protein that promotes growth, differentiation and survival of specific populations of neurons (Levi-Montalcini, 1987) and that also exerts differentiating effects on cells of endocrine origin (Missale *et al.*, 1994). Short-term exposure of bromocriptine-resistant prolactinomas to NGF, both *in vitro* and *in vivo*, results in their long lasting conversion into lactotroph-like cells that once again express the D-2 receptor protein (Missale *et al.*, 1993). The possibility thus exists that short-term treatment with NGF might restore responsiveness to bromocriptine therapy in patients resistant to dopaminergic treatment.

Correspondence: C Missale, PF Spano, Division of Pharmacology, Department of Biomedical Sciences and Biotechnology, University of Brescia, Via Valsabbina 19, 25124 Brescia, Italy
Received 16 June 1995; accepted 8 August 1995

In the present study, we investigated whether or not sequential administrations of NGF and bromocriptine were an effective therapy for athymic mice transplanted with bromocriptine-resistant human prolactinomas.

Cells obtained from one human bromocriptine-resistant prolactinoma were transplanted, s.c. ($10^7$ cells per mouse) into 24 Nu/Nu mice (20 g body weight; Charles River Breeding Laboratories). After development of tumours of $15 \pm 1$ mm$^3$, the mice were divided into four groups. Two groups were treated with saline ($n = 12$) and two groups with NGF (1 $\mu$g g$^{-1}$ body weight, i.v.; $n = 12$) once a day for 5 consecutive days. Fifteen days after the last NGF administration one subgroup of controls ($n = 6$) and one subgroup of NGF-treated mice ($n = 6$) were injected with bromocriptine to determine plasma PRL levels. Tumour size was measured before and after NGF administration during a 40 day follow-up period.

Figure 1 shows the sizes of the transplanted tumours in saline- and NGF-treated mice at the end of the follow-up. Tumours of $600 \pm 40$ mm$^3$ were detectable in saline-treated mice (Figure 1a). The 5 day NGF treatment inhibited tumour growth remarkably so that the tumours grew to only $72 \pm 10$ mm$^3$ (Figure 1b). The complete tumour growth curves for saline- and NGF-treated mice are shown in Figure 2a. The effect of NGF was already evident at the end of treatment and was even more significant after prolonged follow-up. None of the 12 mice injected with NGF died during treatment. In addition, no important side-effects were noted following NGF administration.

Bromocriptine did not modify tumour growth in saline-treated mice (Figure 2b). However, the drug recovered its full activity when injected into mice previously treated with NGF. As a result of bromocriptine administration, the tumour size progressively decreased, completely regressing within 11 days (Figure 2b). Tumours did not reappear after bromocriptine therapy was discontinued (Figures 1c and 2b).

Mice transplanted with prolactinomas had higher plasma PRL levels than normal animals. Analysis of blood samples obtained immediately before and 2 days after beginning bromocriptine administration revealed bromocriptine-induced normalisation of PRL levels in NGF-treated animals ($650 \pm 52$ ng ml$^{-1}$ before bromocriptine and $195 \pm 15$ ng ml$^{-1}$ after bromocriptine administration). Similar results were obtained with two additional human tumours.

Bromocriptine thus recovered both its PRL-suppressing and antiproliferative effects in nude mice grafted with DA-insensitive tumours when they had been pretreated with NGF.

Short-term treatment with NGF thus induces long-lasting

Figure 1 Appearance of human bromocriptine-resistant prolactinomas in athymic mice. Nu/Nu mice transplanted with bromocriptine-resistant human prolactinomas were treated with saline or NGF (1 $\mu g\ g^{-1}$ body weight, i.v.) for 5 days. Fifteen days after the last NGF administration one group of NGF-treated mice was injected with bromocriptine (0.01 $\mu g\ g^{-1}$ body weight; i.p.) for 15 days. (**a**) Tumours in anesthetised saline-treated mice 40 days after the last saline administration. (**b**) Tumours in anesthetised NGF-treated nude mice 40 days after the last NGF administration. (**c**) Regression of tumours by bromocriptine in nude mice pretreated with NGF.

Figure 2 Effects of bromocriptine on tumour growth in saline- and NGF-treated nude mice. Mice transplanted with bromocriptine-resistant human prolactinomas were treated with saline or NGF (1 $\mu g\ g^{-1}$ body weight, i.v.) for 5 days and then with bromocriptine (0.01 $\mu g\ g^{-1}$ body weight; i.p.) for 15 days. (**a**) tumour growth curves in saline (O) and NGF-treated (●) nude mice. From days 36 to 46 tumours completely regressed. Points are the means ± s.e.m. of six mice in each group. $^*P<0.001$ *vs* saline-treated mice; Student's *t*-test. Statistical analysis was applied to points corresponding to individual times.

and full sensitivity to subsequent therapy with bromocriptine in mice transplanted with DA-resistant human prolactinomas. Remarkably, the effects of bromocriptine in this model are similar to those observed in patients with prolactinomas sensitive to the dopaminergic therapy (Cunnah and Besser, 1991; Wood *et al*., 1991).

The present data, obtained in a proper animal model, show the first successful pharmacological treatment of DA-resistant human macroprolactinomas and open the way to development of a new, promising therapy for non-responding patients. It is important that only a few doses of NGF have to be administered to prime refractory patients to respond to bromocriptine, since there are fewer side-effects when NGF is administered acutely. It has been shown, in fact, that a single administration of NGF to healthy human volunteers is not life-threatening and induces only transient, mild to moderate muscle pain (Petty *et al*., 1994).

Therefore, sequential therapy with NGF and bromocriptine appears to be a potential and promising alternative to neurosurgical intervention for patients with DA-resistant prolactinomas.

### References

CUNNAH D AND BESSER M. (1991). Management of prolactinomas. *Clin. Endocrinol*, **34**, 231–235.

DAVIS JRE, SHEPPARD MC AND HEATH DA. (1990). Giant invasive prolactinoma: a case report and review of nine further cases. *Q. J. Med*., **74**, 227–238.

LEVI-MONTALCINI R. (1987). The nerve growth factor 35 years later. *Science*, **237**, 1145–1162.

MISSALE C, BORONI F, LOSA M, GIOVANELLI M, ZANELLATO A, DAL TOSO R, BALSARI A AND SPANO PF. (1993). Nerve growth factor suppresses the transforming phenotype of human prolactinomas. *Proc. Natl Acad. Sci. USA*, **90**, 7961–7965.

MISSALE C, BORONI F, SIGALA S, ZANELLATO A, DAL TOSO R, BALSARI A AND SPANO PF. (1994). Nerve growth factor directs differentiation of the bipotential cell line GH-3 into the mammotroph phenotype. *Endocrinology*, **135**, 290–298.

PELLEGRINI I, ROSOLONJANAHARY R, GUNZ G, BERTRAND P, DELIVET S, JEDYNAK CP, KORDON C, PEILLON F, JAQUET P AND ENJALBERT A. (1989). Resistance to bromocriptine in prolactinomas. *J. Clin. Endocrinol. Metab.*, **69**, 500–509.

PETTY BG, CORNBLATH DR, ADORNATO BT, CHAUDRY V, FLEXNER BT, WACHSMAN M, SINICROPI D, BURTON LE AND PEROUTKA SJ. (1994). The effect of systemically administered recombinant human nerve growth factor in healthy human subjects. *Ann. Neurol.*, **36**, 244–246.

SCHLECHTE JA, SHERMAN BM, CHAPLER FK AND VAN GILDER J. (1986). Long-term follow up of women with surgically treated prolactin-secreting pituitary tumors. *J. Clin. Endocrinol. Metab.*, **62**, 1236–1301.

WOOD DE, JOHNSTON JM AND JOHNSTON DG. (1991). Dopamine, the dopamine D-2 receptor and pituitary tumors. *Clin. Endocrinol.*, **35**, 455–466.